# Complementary traditional Chinese medicine therapy improves survival in patients with metastatic prostate cancer

**DOI:** 10.1097/MD.0000000000004475

**Published:** 2016-08-07

**Authors:** Jui-Ming Liu, Po-Hung Lin, Ren-Jun Hsu, Ying-Hsu Chang, Kuan-Chen Cheng, See-Tong Pang, Shun-Ku Lin

**Affiliations:** a Division of Urology, Department of Surgery, Taoyuan General Hospital, Ministry of Health and Welfare; b Division of Urology, Department of Surgery, Chang Gung Memorial Hospital; c Graduate Institute of Clinical Medical Sciences, College of Medicine, Chang Gung University; d Biobank Management Center of the Tri-Service General Hospital, National Defense Medical Center; e Department of Pathology and Graduate Institute of Pathology and Parasitology, the Tri-Service General Hospital, National Defense Medical Center; f Graduate Institute of Life Sciences, National Defense Medical Center; g Graduate Institute of Food Science and Technology, National Taiwan University; h Institute of Biotechnology, National Taiwan University; i Department of Medical Research, China Medical University Hospital, China Medical University; j Department of Chinese Medicine, Taipei City Hospital, Renai Branch, Taipei City, Taiwan.

**Keywords:** androgen deprivation therapy, complementary therapies, National Health Insurance Research Database, prostate cancer, traditional Chinese medicine

## Abstract

More than 50% of prostate cancer patients have used traditional Chinese medicine (TCM) in Taiwan. However, the long-term clinical efficacy of TCM in prostate cancer patients remains unclear. Here, we investigated the relationship between TCM use and the survival of prostate cancer patients.

A retrospective nationwide cohort study of prostate cancer patients was conducted between 1998 and 2003 using the Taiwan National Health Insurance Research Database. Patients were classified as TCM users or nonusers, and monitored from the day of prostate cancer diagnosis to death or end of 2012. The association between death risk and TCM use was determined using Cox proportional-hazards models and Kaplan–Meier curves.

Of the 1132 selected prostate cancer patients, 730 (64.5%) and 402 (35.5%) were TCM users and nonusers, respectively. The mean follow-up period was 8.38 years, and 292 (25.8%) deaths were reported. TCM users had a decreased mortality rate (21.9%) compared with nonusers (32.8%). A lower death risk was observed with longer TCM use, especially in patients who used TCM for ≧200 days (adjusted hazard ratio [aHR] 0.61, 95% confidence interval [CI] 0.44–0.84). TCM users with metastatic prostate cancer had a significant lower HR than nonusers (aHR 0.70, 95% CI 0.51–0.95). Chai-Hu-Jia-Long-Gu-Mu-Li-Tang was the most significant TCM formulae for improving survival in metastatic prostate cancer (aHR 0.18, 95% CI 0.04–0.94).

The result suggested that complementary TCM therapy might be associated with a reduced risk of death in metastatic prostate cancer patients.

## Abstract 2

Supplemental Digital Content is available in the text

## 1 Introduction

Prostate cancer is the most common cancer in men, and has increased in modern society, especially in people aged >65 years.^[1]^ The incidence of prostate cancer rapidly increased in the recent few years in Taiwan (from 16.57 per 100,000 in 2000 to 40.56 in 2012).^[2]^ The different types of treatment for distinct prostate cancer stages include radical prostatectomy, radiation therapy, hormone therapy, and chemotherapy,^[3]^ but prostate cancer remains as one of the leading causes of cancer-related deaths in Taiwan. The incidence of prostate cancer-related death has also increased recently (5.59 per 100,000 in 2000 to 10.17 in 2012).^[4]^ Therefore, patients and caregivers often consider complementary and alternative medicine as another treatment choice.^[5]^ In Taiwan, traditional Chinese medicine (TCM) is the main form of complementary and alternative medicine. Lin et al demonstrated that an overall 52.6% of prostate cancer patients had used TCM in their 6-year cohort study. They also found a trend of increased TCM use among prostate cancer patients under the National Health Insurance (NHI), especially in cancer-specific TCM visits.^[6]^

According to in vitro and in vivo studies, TCM might be beneficial for prostate cancer patients by inhibiting the invasion of cancer cells, inducing apoptosis, suppressing prostate cancer-dependent angiogenesis, and down-regulating human androgen receptors.^[7]^ However, there is a lack of large-scale studies to verify the long-term outcomes of TCM. Here, we used nationwide data to estimate the survival benefit after TCM treatment for patients with prostate cancer. To the best of our knowledge, this was the first study to date involving the largest cohort and longest follow-up period to investigate such an issue.

## 2 Materials and methods

### 2.1 Longitudinal health insurance database

We designed a retrospective cohort study using 15 years data (from January 1, 1998 to December 31, 2012) of the Longitudinal Health Insurance Database 2005 (LHID2005), a subdataset of the National Health Insurance Research Database (NHIRD). The NHIRD is a national-scale research library with recorded medical and demographic information from >99% of the Taiwan population for >20 years, and >2000 studies have been currently published using the NHIRD.^[8]^ The LHID2005 randomly selected 1 million people from the entire 23 million insurers of the NHIRD in 2005, and the demographic factors were similar between people in the LHID2005 and origin NHIRD.^[9]^ The NHIRD included detailed outpatient and inpatient medical records, such as visiting or admission date, clinical diagnosis according to the International Classification of Diseases, Ninth Revision, Clinical Modification (ICD-9-CM) codes, surgical procedure, and drug information (i.e., prescription date, duration, and dosage). Moreover, LHID2005 provides uninterrupted longitudinal tracking for every patient. Therefore, LHID2005 is suitable for long-term research about cancer and chronic diseases.^[10]^ The study was reviewed and approved by the institutional review board of the Chang Gung Memorial Hospital, Linkou, Taiwan (CGMH IRB 103-3238B).

### 2.2 Study participants

**Figure 1:**
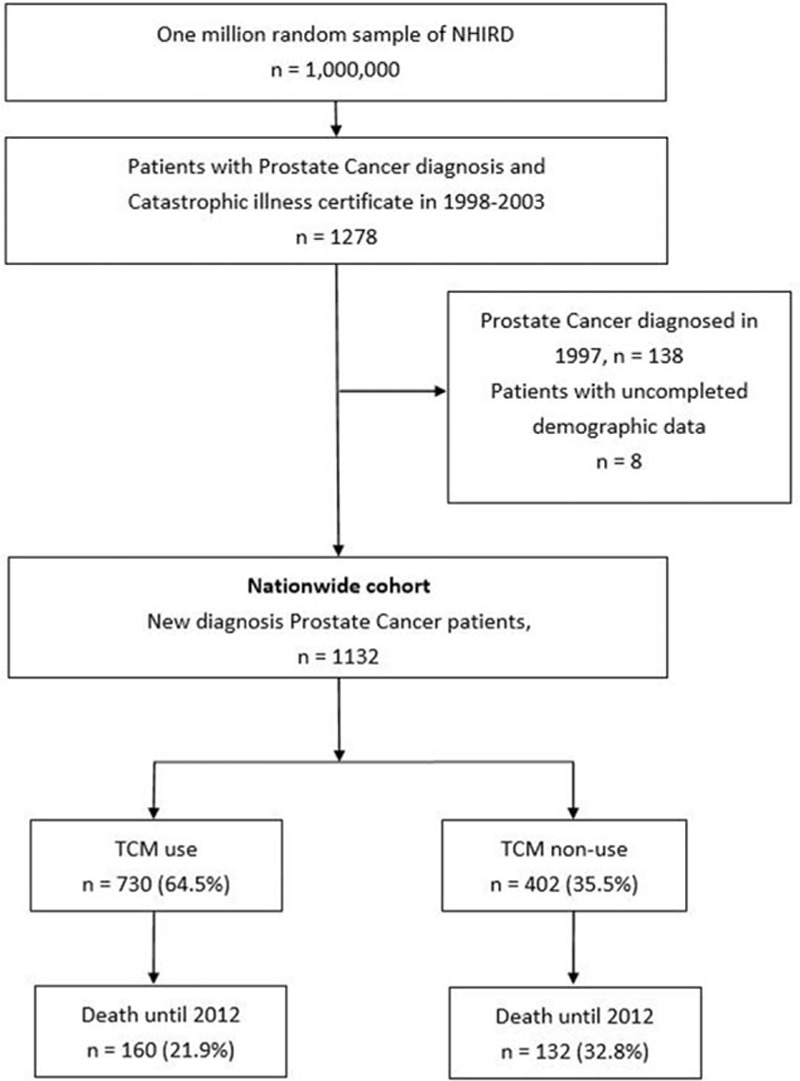
Flowchart of the patient enrollment procedure from one million longitudinal health insurance database. We identified patients with diagnosis of prostate malignant neoplasm by ICD-9 code (185) in Taiwan between 1998 and 2003, and patients were divided in to TCM use (n = 730) and TCM nonuse (n = 402). ICT-9 = The International Classification of Diseases, Ninth Revision; TCM = traditional Chinese medicine.

The flowchart of patient enrollment is shown in Fig. 1. Patients who were diagnosed with prostate cancer (ICD-9-CM code 185) and who obtained catastrophic certification were selected from the LHID2005 database.^[11]^ Catastrophic certification was reviewed and issued by the Ministry of Health and Welfare; the audit process included diagnosis by a urology specialist and chart revision by senior physicians. Furthermore, the pathology and imaging report would also be censored. Therefore, patients who had catastrophic certification of prostate cancer could be definitely confirmed to have prostate cancer.

A total of 1278 patients who were diagnosed with prostate cancer and issued catastrophic illness certificates were selected between 1998 and 2003. We excluded 138 patients who were diagnosed with prostate cancer in 1997 and 8 patients with uncompleted demographic data. Finally, 1132 patients with prostate cancer were enrolled in this study. We use the following steps to divided prostate cancer patients into TCM user and TCM nonuser groups. First, we obtained all outpatient medical data of prostate cancer patients from the NHIRD file “Ambulatory care expenditures by visits,” which contained detailed outpatient medical information including medical divisions, specialist of physician, date, and hospital or clinic. Second, we excluded all outpatient records before prostate cancer diagnosis and isolated the medical information of “traditional Chinese medicine divisions” as TCM groups. Patients who did not have Chinese medicine outpatient records were classified as “non-TCM” groups. Third, we link outpatient data with files “Details of ambulatory care orders,” which contain the information of every outpatient treatment, including drug name and dosage regimens. Fourth, we calculated each patient's Chinese medical treatment time and dose of herbal formulae. The patients were divided into 2 groups: TCM users (730, 64.5%) and TCM nonusers (402, 35.5%). All medical diagnoses, surgical procedures, and medications were completely recorded during the follow-up period.

### 2.3 Adjustment of covariates

We classified patients into the following 4 age groups: <60 years, 60 to 70 years, 70 to 80 years, and >80 years. We classified the area of Taiwan into 4 groups: low urbanization, moderate urbanization, high urbanization, and very high urbanization. We also classified insurance payments, in New Taiwan Dollars (NT$), into the following 4 groups: dependent (no constant income), NT$ 1 to 19,999; NT$ 20,000–39,999, and >NT$ 40,000. The Charlson comorbidity index scores were categorized into the following 4 groups and were used to measure comorbidity from the NHIRD: ≦2, 2 to 4, 4 to 6, and >6. To assess the dose–response relationship between TCM use and the reduction of the risk of death, we divided TCM users into 3 groups according to the TCM treatment duration: <50 days (n = 241), 50 to 200 days (n = 240), and ≧200 days (n = 249). We included comorbidities related to prostate cancer according to previous studies in the literature,^[12]^ including diabetes mellitus, chronic kidney disease, cerebrovascular accident, coronary heart disease, liver cirrhosis, and hypertension. The HR with 95% CI of the mortality in national prostate cancer cohort by different adjust model was listed in Supplementary File 1.

The clinical stage of prostate cancer was not recorded in the NHIRD; therefore, we used the initial treatment to assess the clinical stage of prostate cancer. Patients who received radical prostatectomy—radiation therapy—were assigned to the localized or locally advanced prostate cancer group. Patients who initially received androgen deprivation therapy (ADT) were assigned to the metastatic prostate cancer group. Patients who received chemotherapy were assigned to the castration-resistant prostate cancer group.

### 2.4 Traditional Chinese medicine

We included all TCM records of prostate cancer patients during the study period. In Taiwan, only a licensed TCM physician could apply for payment from NHI, and medical records would be censored strictly to ensure the quality of medical service.

The TCM formulae covered by NHI were approved by the Ministry of Health and Welfare, Taiwan. To meet the Good Manufacturing Practice standards, all these formulae were required and all ingredients were clearly declared.

### 2.5 Study outcome

The main outcome was all-cause mortality; the death was defined as not only discharge from the hospital due to death but also withdrawal from the NHI insurance. The definition of death was adopted from a previous study.^[13]^ The follow-up period was calculated from the day of prostate cancer diagnosis to death or end of study (December 31, 2012).

### 2.6 Statistical methods

We use logistic regression to determine factors related to TCM use among prostate cancer patients. We also performed Cox proportional regression models to analyze adjusted HRs (aHRs) and accompanying 95% CIs after adjusting for the aforementioned variables; the significance level (α) was set at 0.05. Furthermore, Kaplan–Meier curves were used to show the difference in survival between the TCM users and nonusers. The statistical software package SAS (version 9.4, SAS Institute, Cary, NC) was used for data analysis.

## 3 Results

From 1998 to 2003, 1132 newly diagnosed prostate cancer patients were enrolled in this study after excluding patients with uncompleted demographic data; 730 (64.5%) and 402 (35.5%) of the included patients were TCM users and nonusers, respectively. The incidence of prostate cancer was 32.14 per 100,000 males per year, and the prevalence was 217.73 per 100,000 males in 2003. The mean follow-up duration was 8.38 years. A total of 292 prostate cancer patients died during the study period (mortality rate 25.6%); of these patients, 160 (21.9%) and 132 (32.8%) patients were TCM users and nonusers, respectively (Fig. 1). NHIRD contained TCM treatment record including herb formulae, acupuncture, and massage techniques. In the present study, 79.5% prostate cancer patients received herbal medicine treatment; acupuncture and massage techniques accounted for 20.5%.

**Figure 2:**
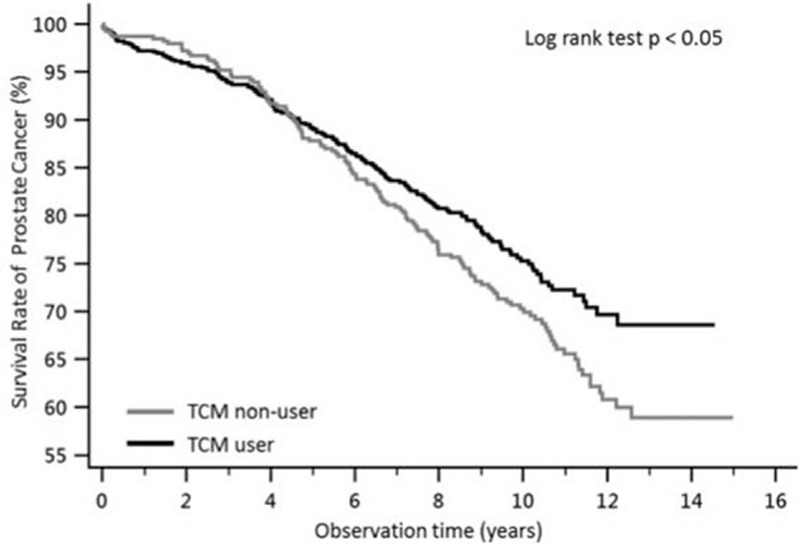
Survival curve of patients with prostate cancer according to traditional Chinese medicine use. The survival rate was significant different between TCM uses and TCM nonusers in Kaplan–Meier estimator, and the *P* value of log-rank test <0.05. TCM = traditional Chinese medicine.

**Figure 3:**
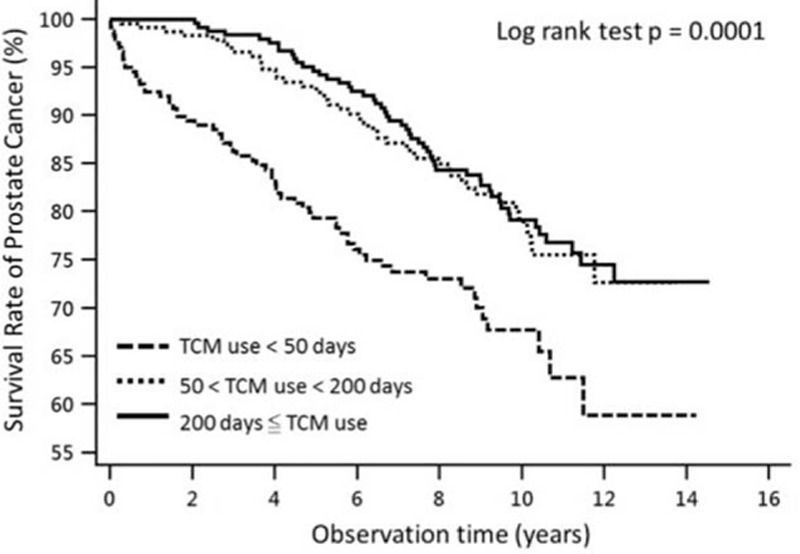
Survival curve of patients with prostate cancer according to traditional Chinese medicine duration. The survival rate was significant different between 3 groups in Kaplan–Meier estimator, and the *P* value of log-rank test was 0.0001.

The Kaplan–Meier survival curve for prostate cancer patients according to TCM use is shown in Fig. 2. There mortality rate in TCM users was significant lower compared to nonusers. TCM users had a significantly higher survival rate (80.8% in 8 years, 75.0% in 10 years, and 68.6% in 12 years) than nonusers (76.9% in 8 years, 69.9% in 10 years, and 60.8% in 12 years). We also illustrated the survival curve of patients with prostate cancer according to TCM duration in Fig. 3. The survival rate of prostate cancer patients was significantly different among the TCM duration groups (*P* < 0.0001).

The demographic characteristics, which affected the usage of TCM in prostate cancer patients, are shown in Table 1. Being of older age (70–80 years and more than 80 years) might reduce patients’ wishes to seek TCM treatment. The insured amount, comorbidity, and the Charlson comorbidity index showed no significant influence on TCM use.

**Table 1:**
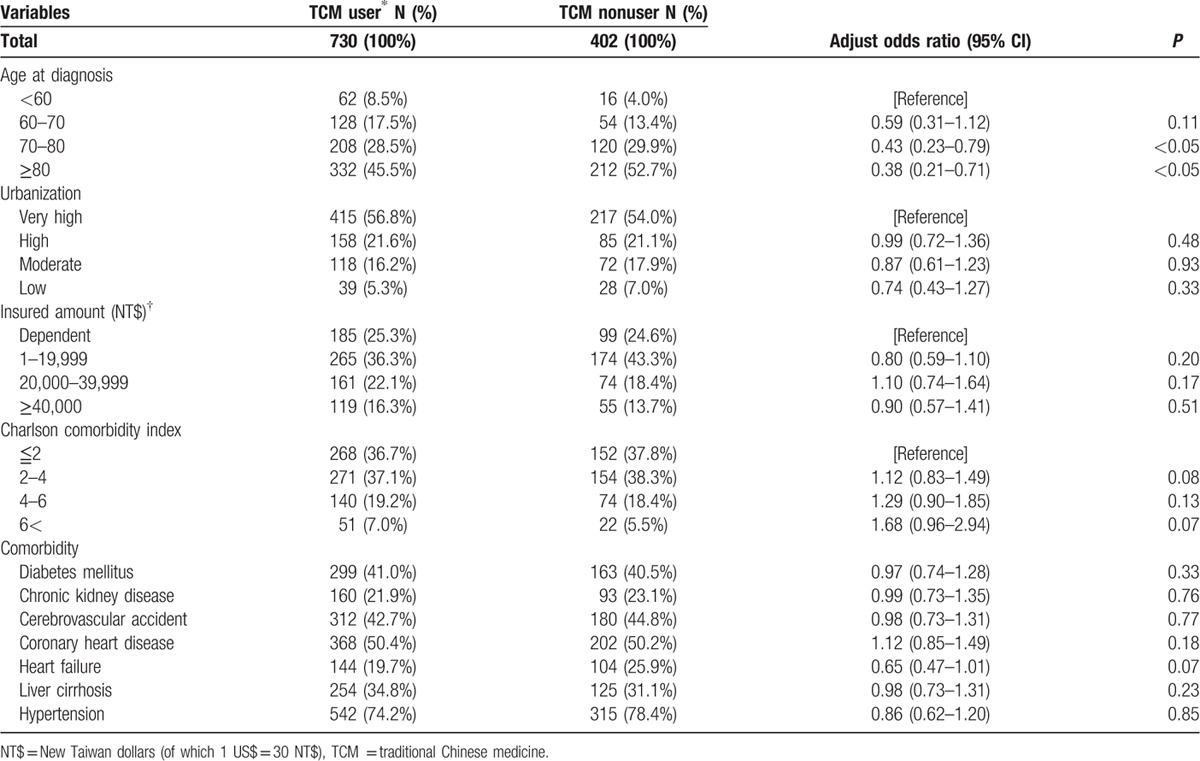
Demographic and medical characteristics of prostate cancer patients according to use of traditional Chinese medicine.

The aHRs for mortality in prostate cancer patients is shown in Table 2. Compared with TCM nonusers, patients who use TCM for more than 50 days have a lower risk of death. The risk of death in patients who used TCM for 50 to 200 days and ≧200 days decreased by 31% (aHR 0.69, 95% CI 0.50–0.97, *P* = 0.03) and 39% (aHR 0.61, 95% CI 0.44–0.84, *P* < 0.001), respectively. Furthermore, old age, low amount of insurance, high Charlson comorbidity index, liver cirrhosis, and hypertension were significantly related to an increase risk of death from prostate cancer.

**Table 2:**
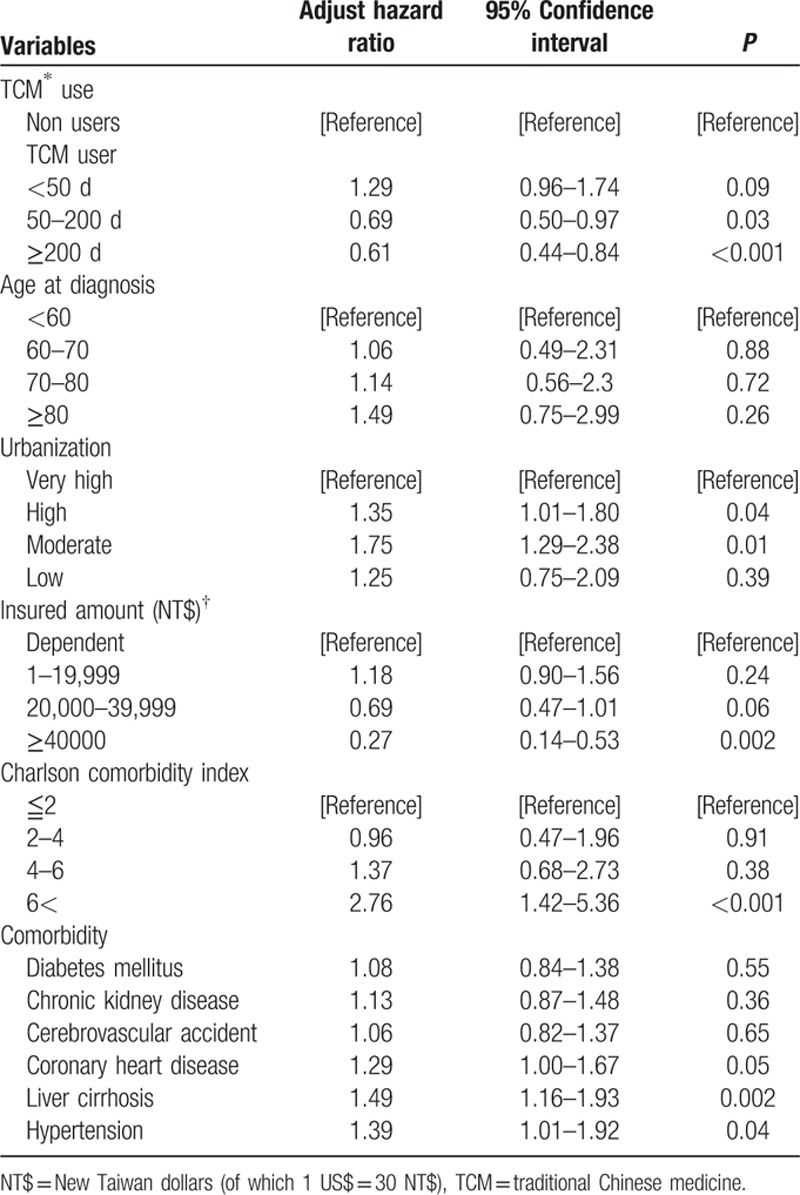
Adjust hazard ratio with 95% confidence interval of the mortality in National Prostate Cancer Cohort.

The mortality rate of different prostate cancer groups is shown in Table 3. Approximately, 37.3%, 61.2%, and 1.5% of TCM users had localized or locally advanced, metastatic, and castration-resistant prostate cancer, respectively. TCM users in the metastatic prostate cancer group had a significant better survival rate compared with TCM nonusers (aHR 0.70, 95% CI 0.51–0.95, *P* = 0.02). The mortality risk in the localized or locally advanced and castration-resistant prostate cancer groups was not significantly different between TCM users and nonusers.

**Table 3:**
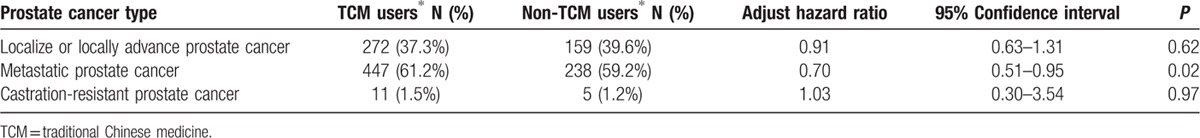
Mortality in National Prostate Cancer Cohort, analyzed by multivariable Cox proportional-hazards regression model and 95% confidence intervals.

The top 10 TCM formulae, which might influence the survival rate among metastatic prostate cancer patients, are shown in Table 4. The TCM formulae, Chai-Hu-Jia-Long-Gu-Mu-Li-Tang, had the most significant improvement in the survival rate of metastatic prostate cancer patients (aHR 0.18, 95% CI 0.04–0.94, *P* =  0.04). The following formulae also improved the survival rate: Suan-Zao-Ren-Tang (aHR 0.28, 95% CI 0.08–0.97, *P* =  0.04), Ban-Xia-Xie-Xin-Tang (aHR 0.34, 95% CI 0.12–0.95, *P* = 0.04), and Ba-Wei-Di-Huang-Wan (aHR 0.39, 95% CI 0.20–0.78, *P* = 0.02).

**Table 4:**
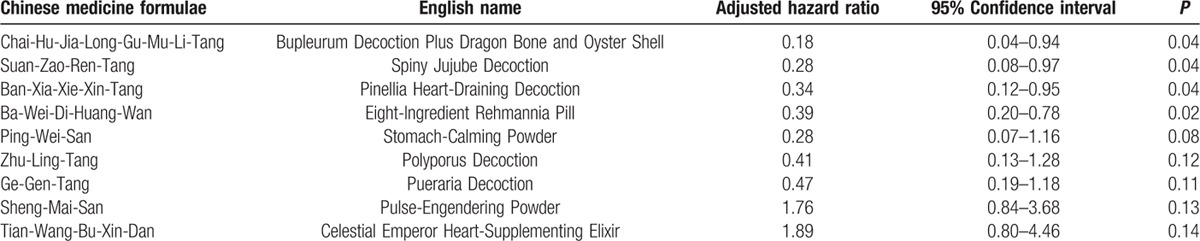
Adjusted Cox proportional-hazards ratio of the commonly used Chinese medicine formulae in metastatic prostate cancer.

## 4 Discussion

To the best of our knowledge, this is the first and largest study to evaluate the relationship between TCM use and survival of prostate cancer patients. We enrolled over 1000 patients who were followed up for an average of 8 years in a retrospective nationwide cohort study. Prostate cancer patients who received TCM treatments for >50 days had a lower mortality risk compared with nonusers. TCM users with metastatic prostate cancer were observed to have the most significant improvement in survival compared with TCM nonusers. The Chai-Hu-Jia-Long-Gu-Mu-Li-Tang, Suan-Zao-Ren-Tang, Ban-Xia-Xie-Xin-Tang, and Ba-Wei-Di-Huang-Wan were the most likely TCM formulae to reduce the risk of death in metastatic prostate cancer patients. In the present large-scale study, we were able to provide both the evidence for the benefits of TCM and an analysis of the prescription of TCM. The study result may help us to correlate clinical practice with basic research.

The potential mechanisms by which TCMs act against cancer have been established in previous studies, such as modulation of immunity,^[14]^ inflammation,^[15]^ osteogenesis,^[16]^ hematopoiesis,^[17]^ and neuroprotection.^[18]^ According to the results of this study, some TCMs that were found to be beneficial for prostate cancer treatment (i.e., Chai-Hu-Jia-Long-Gu-Mu-Li-Tang and Ba-Wei-Di-Huang-Wan) improved the survival rate of prostate cancer patients in long-term follow-up.

In the present study, the TCM formula, Chai-Hu-Jia-Long-Gu-Mu-Li-Tang, had the most significantly improved survival rate in metastatic prostate cancer patients. Chai-Hu-Jia-Long-Gu-Mu-Li-Tang was used to relieve symptoms of hypogonadism, including insomnia, hot flushes, and erectile dysfunction, but did not lead to changes in serum testosterone levels.^[19]^ Furthermore, Ha et al^[20]^ demonstrated that extracts from Chai-Hu-Jia-Long-Gu-Mu-Li-Tang had inhibitory effects on tumor-specific matrix metalloproteinases (MMP)-2 and MMP-9 activities, which are associated with tumor recurrence and progression in prostate cancer.^[21]^ According to the literature, the major components of Chai-Hu-Jia-Long-Gu-Mu-Li-Tang also have anticancer effects. For example, baicalin and baicalein are extracted from Radix Scutellariae, which inhibits prostate cancer growth,^[22]^ induces apoptosis,^[23]^ and reduces free radicals.^[24]^ Saikosaponin-d, extracted from Bupleuri Radix, inhibited human prostate cancer cells by inducing apoptosis and blocking the cell cycle.^[25]^ 6-Shogaol, a component of ginger, restrained the growth of prostate cancer cells both in vitro and in vivo by inhibiting signal transducer and activator of transcription 3 (STAT3) and nuclear factor kappa-light-chain-enhancer of activated B cell (NF-κB) signaling.^[26]^

Ba-Wei-Di-Huang-Wan also showed a potential to reduce the mortality rate (aHR 0.39, 95% CI 0.20–0.78). Ba-Wei-Di-Huang-Wan was widely used for treating lower urinary tract symptoms of benign prostate hyperplasia, such as nocturia or incomplete bladder emptying.^[27]^ Ba-Wei-Di-Huang-Wan can reduce osteoporosis, a common side effect of ADT, and maintain trabecular bone mass and bone mineral density by activating bone metabolism in castrated mice.^[28,29]^ Furthermore, it can reduce fracture in prostate cancer patients. Ba-Wei-Di-Huang-Wan was not shown to increase serum testosterone level or prostate volume in animal studies.^[30,31]^

Additionally, we found that some TCM formulae that were rarely used to treat prostate cancer, such as Suan-Zao-Ren-Tang, Ping-Wei-San, and Ban-Xia-Xie-Xin-Tang, may improve the survival rate of prostate cancer patients in the present study. Further studies may be needed to explore the mechanism of these TCMs for improving the survival rate in prostate cancer patients. In contrast, several complementary and alternative medicines, which have been well-established for treating prostate cancer, were not widely used in Taiwan. For example, Rhizoma Curcumae Longae had anticancer effects by inhibiting prostate cancer cell growth^[32]^ and vascular endothelial growth factor, and reducing angiogenesis of cancer.^[33]^ It could also play a role of as a chemosensitizer and radiosensitizer for tumor treatment.^[34]^ Furthermore, Rhizoma Curcumae Longae had a better anticancer effect when combined with 5-fluorouracil and paclitaxel in a previous study.^[35]^

All TCM records of prostate cancer patients were included in our study after the initial diagnosis of prostate cancer. This was because patients would visit the TCM clinic for prostate cancer symptoms or side effects of their treatment. Therefore, the rate of TCM use was higher in this study compared with the rate in a previous study ^[6]^; however, we were able to minimize observer bias by the different diagnosis methods for TCM and modern medicine.

In our study, the 10-year mortality rate was 25.6%, which was lower than a previous study.^[36]^ The lower mortality rate could be due to the application of a strict definition of mortality (i.e., only patients who were discharged from the hospital due to death and withdrawn from NHI). Although this definition might lead to an underestimation of mortality, it could lead to a better indication of the research outcome.

### 4.1 Limitations

There are several limitations in this study. Firstly, some data, such as prostate-specific antigen (PSA) level, stage of prostate cancer, and tumor grade, were not available from the NHIRD database. Furthermore, it was difficult to clarify the severity of cancer. The Surveillance, Epidemiology, and End Results database also recently excluded all PSA data by the National Cancer Institute because of the inaccurate PSA values and misinterpretation of PSA variables.^[37]^ Although we did not have complete clinical information, the prostate cancer patients were divided into 3 groups according to types of prostate cancer treatment, which helped us define the severity of prostate cancer and further demonstrate the effects of TCM use in these groups. Secondly, the TCM use could have been underestimated because the NHIRD database only included Chinese herbal medicine and acupuncture therapy prescriptions by licensed physicians. Other alternative Chinese medicines, such as natural and folk medicines, or exercise therapy including Tai-Qi, were not included in this study, because we were unable to incorporate these data in our study. Thirdly, there might be differences in the treatment of prostate cancer according to the TCM physician; however, their treatment practices would comply with treatment guidelines. Finally, this was a retrospective observational study; we could only calculate the correlation between mortality risk by using TCM formulae, and we are still looking for more clinical trials to confirm real causality of different prescriptions.

## 5 Conclusions

On the basis of the results of this retrospective cohort study, adjunctive TCM therapy with ADT might improve the survival of metastatic prostate cancer patients. Therefore, TCM treatment can be used as a component of cancer treatment in these patients.

## Supplementary Material

Supplemental Digital Content
