# Supplementary material for: Complementary traditional Chinese medicine therapy improves survival in patients with metastatic prostate cancer
Source: Medicine (Baltimore). 2016 Aug 7;95(31):e4475. doi: 10.1097/MD.0000000000004475 (PMC4979842; doi:10.1097/MD.0000000000004475)
Supplement: Supplemental Digital Content [file medi-95-e4475-s001.docx]

**Supplementary File 1.** Hazard Ratio with 95% Confidence Interval of the Mortality in National Prostate Cancer Cohort by Different Adjust Model

| Hazard Ratio  (95% Confidence Interval) | Model 1 | Model 2 | Model 3 | Model 4 | Model 5 |
| --- | --- | --- | --- | --- | --- |
| TCM^a^ user < 50 Days | 1.23(0.92-1.65) | 1.25(0.95-1.54) | 1.26(0.93-1.67) | 1.34(0.97-1.82) | 1.29(0.96-1.74) |
| TCM user 50-200 Days | 0.64(0.47-0.88) | 0.67(0.48-0.82) | 0.65(0.49-0.92) | 0.71(0.52-0.95) | 0.69(0.50-0.97) |
| TCM user≧200 Days | 0.57(0.43-0.78) | 0.56(0.42-0.76) | 0.61(0.48-0.80) | 0.65(0.43-0.74) | 0.61(0.44-0.84) |

**Model 1** Crude hazard ratio of the mortality in national prostate cancer cohort between TCM users and non users

**Model 2** Hazard ratio was adjusted by age at diagnosis.

**Model 3** Hazard ratio was adjusted by age at diagnosis and Charlson comorbidity index.

**Model 4** Hazard ratio was adjusted by age at diagnosis, Charlson comorbidity index, urbanization, and insured amount.

**Model 5** Hazard ratio was adjusted by age at diagnosis, Charlson comorbidity index, urbanization, insured amount, and comorbidity.

^a^ TCM represent traditional Chinese medicine.
